# The complexity of molecular processes in osteoarthritis of the knee joint

**DOI:** 10.1515/med-2020-0402

**Published:** 2020-04-21

**Authors:** Paweł Łęgosz, Sylwia Sarzyńska, Łukasz Pulik, Daniel Kotrych, Paweł Małdyk

**Affiliations:** Department of Orthopaedics and Traumatology, 1st Faculty of Medicine, Medical University of Warsaw, Warsaw, Poland; Department of Orthopaedics, Traumatology and Orthopaedic Oncology, Pomeranian Medical University in Szczecin, Szczecin, Poland

**Keywords:** osteoarthritis, metalloproteinases, interleukins, total knee arthroplasty, cartilage

## Abstract

Osteoarthritis (OA) is a common medical problem leading to chronic pain and physical disability among the world’s population. Analyzing the molecular background of the degenerative arthritis creates the potential for developing novel targeted methods of treatment. Fifty samples of meniscus, anterior cruciate ligaments (ACLs) and articular surfaces were collected from patients who underwent total knee arthroplasty in 2016. Enzyme-linked immunosorbent assay was used to assess the levels of interleukin (IL)-1β, IL-6, tumor necrosis factor (TNF), transforming growth factor-β1 and LUMINEX for MMP-1, MMP-2, MMP-3, MMP-9 and MMP-13. The collected data were correlated with the severity of radiological OA, demographic data and clinical scales. Strong positive correlations in the concentration of metalloproteinases and proinflammatory cytokines, TNF-α (MMP-2 and MMP-13) and IL-6 (MMP-13), were identified. MMP-13 had a positive correlation with the concentration of MMP-1, MMP-2 and MMP-9. Negative correlation coefficient exists between clinical conditions measured with the Western Ontario and McMaster Universities Osteoarthritis Index scale and the level of TNF-α and MMP-1. The TNF-α concentration was lower in the cartilage of the articular surface among patients who took non-steroidal anti-inflammatory drugs periodically. The decrease in MMP-2 in the cartilage of the articular surface corresponded with the severity of radiological OA on the Kellgren–Lawrence scale. Current treatment methods for OA do not stop disease progression. Identifying signaling pathways and molecular particles engaged in OA and their correlations with the patient’s clinical condition brings new therapeutic possibilities.

## 1 Introduction

Osteoarthritis (OA) is a common medical problem resulting in chronic pain and physical disability among the world’s population. Two hundred and fifty million people are estimated to suffer from knee OA worldwide [1]. The condition is a considerable burden in both social and economic aspects [2]. The disease can develop in any joint; however, it usually affects knees, hips, small joints in the hands and feet, talocrural region and the vertebral column [3]. Among the most frequent risk factors identified are age, joint instability or improper joint alignment, obesity, peripheral neuropathies, muscle weakness and crystal deposition diseases of the joints [3]. The etiology of the disease has not been fully explained yet, and its therapy is still a challenge. Investigating molecular aspects of the condition is therefore of great importance and may, in the future, help in developing novel targeted therapeutic strategies to prevent the occurrence and progression of degenerative lesions.

By definition, OA is characterized by metabolic, structural and functional alterations within the whole joint and closely surrounding tissues — pathological lesions are therefore localized in the articular cartilage, subchondral bone, synovium, as well as ligaments and the adjacent muscle tissue [4,5]. The influence of both the local inflammatory process and biomechanical factors on the course of OA is considered to be substantial and is of great significance in terms of disease progression, course and pain intensity [6,7].

The presence of inflammation inhibits the expression of numerous genes involved in the phenotypic differentiation of chondrocytes, which negatively affects their metabolism, transformation and regeneration, hence the progression of the disease [8]. The cartilage tissue becomes damaged due to mechanical stress and joint overstrain [4]. Activation of the extracellular matrix (ECM) receptors, which are present on the chondrocyte surface and are stimulated under the influence of mechanical overload, leads to increased synthesis of proinflammatory cytokines and chemokines as well as ECM-degenerating enzymes [4,9,10]. These include aggrecanases, metalloproteinases as well as serine and cysteine proteases [11]. A considerable increase in the synthesis of mediator molecules, such as interleukin (IL)-1β, tumor necrosis factor alpha (TNF-α) and IL-6, is observed in the course of OA [12,13,14]. It is considered that mainly these molecules, secreted by the injured chondritic, bone and synovial cells, secondarily stimulate the production of other transmitters that exert proinflammatory and catabolic effects on joint structures [8]. Thus, in addition to the aforementioned, the following particles are found within the blood plasma and synovial fluid of patients suffering from OA: products of arachidonic acid metabolism (PGE2 and LTB4), IL-1, IL-8, IL-6, IL-15, IL-17, IL-18, IL-21, leukemia inhibitory factor (LIF) and metalloproteinases, particularly MMP-1, MMP-3, MMP-8 and MMP-13 [8,15]. The increased metalloproteinase synthesis is caused to a large extent by IL-1 and TNF-α. TNF-α enhances both the inflow and adhesion of the inflammatory cells to the site of inflammation. It is important in terms of initiation of angiogenesis as well [16]. In turn, IL-1β, the increased concentrations of which are observed in both the articular cartilage and synovial fluid of patients with OA, influences the synthesis of IL-6, LIF, IL-17 and IL-18. The molecule’s role in intensifying the expression of metalloproteinases, a disintegrin and metalloproteinase with thrombospondin motifs (ADAMTS) and many other enzymes that exert catabolic effects on joint structures has been proved as well [17,18]. The availability of studies that include measurements of the concentrations of proinflammatory factors that are engaged in the etiology of OA and were collected directly from the joint is significantly lower. Liu et al., [19] in their study investigating the role of TLR-2/NF-κB signaling pathway, assessed the levels of proinflammatory particles within the cartilage of people affected by OA (231 patients who underwent total knee arthroplasty [TKA]) and the control group (cartilage from amputated patients – 198). Measurements were performed using quantitative reverse transcription polymerase chain reaction, immunohistochemistry and Western blotting (the expression of TLR-2, NF-κB and MMP-13) as well as enzyme-linked immunosorbent assay (ELISA; associated proinflammatory cytokines). The results indicated an increase in TLR-2, NF-κB, phosphorylated NF-κB, MMP-13, IL-1, IL-6 and TNF-α concentrations in the OA group compared to those of the control group.

The presence of molecules aggravates the damage of the cartilage and leads to the release of microcrystals, cartilage-bone elements and ECM degradation products, including collagen, to the joint cavity. The components of disintegrated ECM proteins, including molecules of collagen and fibronectin, secondarily stimulate further ECM decomposition by inducing proinflammatory cytokines, chemokines and metalloproteinases [5].

All the aforesaid factors contribute to the imbalance between degenerative and regenerative processes within cartilage tissue, leading to the progression of arthropathy.

The pathomechanisms of lesions observed within menisci and ligaments of the knee joint in patients with OA resemble aberrations of the joint cartilage [5].

The aim of this study was the assessment of molecular alterations occurring within joint cartilage, anterior cruciate ligament (ACL) and meniscus collected from patients suffering from OA who underwent TKA.

## 2 Methods

Fifty patients who underwent TKA in 2016 due to primary OA of the knee joint were included in the study. The patients were diagnosed using the criteria set forth by the American College of Rheumatology. The permission to run the study was granted by the Bioethics Committee of Warsaw University of Medicine (KB/12/2016). All the patients signed the informed consent form for participation in the study before undergoing surgery. The main inclusion criteria were idiopathic OA of knee joint of at least two severities on the Kellgren–Lawrence scale and qualification for TKA. The exclusion criteria were as follows: knee joint destruction other than idiopathic degeneration (e.g., post-traumatic degeneration, rheumatoid arthritis and hemophilia); co-morbidities with proven impact on worse TKA prognosis or exacerbating inflammatory processes (psychiatric disorders, diabetes mellitus and grade III obesity); severity of lesions below grade II according to the Kellgren–Lawrence scale; hip and/or ankle disease that could significantly affect the rehabilitation process and impact lower extremity axis; and serum inflammation parameters (C-reactive protein [CRP]) above normal limits.

### 2.1 Data collection and the procedure

During the study, the following demographics and information were collected: the patient’s sex, age, duration of pain, family history, body mass index (BMI), the use of non-steroidal anti-inflammatory drugs (NSAIDs). The Kellgren–Lawrence grading scale was used to assess the severity of radiological changes in OA evaluated by plain radiographs taken just before the procedure. Before the procedure, each patient completed a self-administered clinical scale: the Western Ontario and McMaster Universities Osteoarthritis Index (WOMAC), the Knee injury and Osteoarthritis Outcome Score (KOOS), the Visual Analog Scale (VAS), the Knee Society Score (KSS), Hospital for Special Surgery Knee Score and The Short Form (36) Health Survey (SF-36). Immunoenzymatic measurements of the following cytokine concentrations (ELISA) were used in the analysis: IL-1β, IL-6, TNF, transforming growth factor beta 1 (TGF-β1) and selected matrix metalloproteinases (LUMINEX): MMP-1, MMP-2, MMP-3, MMP-9 and MMP-13, and they were correlated with the patients’ demographic data. Each subject underwent knee joint arthroplasty using a medial parapatellar approach.

### 2.2 Sample collection and preparation

Fifty samples of meniscus, ACLs and articular surfaces were collected from patients. Samples were collected during TKA at subsequent stages of joint preparation (articular surface of tibia, meniscus and ACL) and were placed in sterile containers. Immediately after surgery, the samples were frozen and stored at −80°C for further analyses. Each sample of tissue was homogenized in TissueLyser Bead Homogenizer (Qiagen, USA) and centrifuged (10,000 rpm for 10 min, 4°C). The supernatants were collected and frozen at −80°C until analysis.

#### 2.2.1 Cytokine assays

The evaluation of concentrations of cytokines, including IL-1β, TNF-α and TGF-β1, was carried out by ELISA using commercially available kits purchased from R&D Systems, Inc. (Minneapolis, MN, USA) according to the manufacturer’s instructions. The concentrations of cytokines were determined with a microplate reader Bio-Tek Power Wave XS spectrophotometer (Bio-Tek Instruments, USA).

#### 2.2.2 Multiplex assay

The concentrations of MMP-1, MMP-2, MMP-3, MMP-9 and MMP-13 in tissue homogenates were assessed using a Magnetic Luminex Performance Assay (R&D Systems, Inc.) 5-Plex Panel. Multiplexes were run on a LABScanTM 100 platform (Luminex Corp., Austin, TX, USA) equipped with Luminex^®^ 100 IS software. Tissue homogenates were incubated with antibody-coated microspheres, which bind to specific MMPs. Microsphere–MMP complexes were washed and incubated with biotinylated MMP antibodies, which bind to MMPs present on the microspheres. A final incubation was performed in which phycoerythrin-labeled streptavidin was allowed to bind to biotinylated MMP antibodies present on microspheres. Microspheres were then loaded into a LABScanTM 100 analyzer, which quantifies the amount of phycoerythrin fluorescence present on each of the distinct microsphere groups. At least 50 individual microspheres were counted for each MMP, and the median fluorescence intensity was used for subsequent calculations.

#### 2.2.3 Total protein concentration

Total protein concentration in supernatants was measured at 562 nm on a Bio-Tek Power Wave XS spectrophotometer (Bio-Tek Instruments), using bicinchoninic acid (BCA) Protein Assay Reagent (Pierce, Holland).

Results were presented as an absolute ratio: protein concentration/total protein concentration (×10^−9^).

### 2.3 Statistical analysis

Continuous parameters were characterized by means of the range, mean, standard deviation and median with the lower value (25%) and the upper quartile (75%) in the distribution.

The medians of the continuous variables of the independent groups in the study were compared by means of the *U* Mann–Whitney test or the Kruskal–Wallis test by the ranks in conjunction with Tukey’s *post hoc* test. In order to compare the correlations between quantitative variables, Pearson’s correlation was used. Values below the lower limit of detection (LOD) were substituted with ½ the value of the LOD. In multifactorial modeling of the data, the linear regression and, the stepwise elimination and the logistic regression method were used in order to obtain significant parameters of the model. From this part of the analysis, values below the LOD were eliminated in order to avoid inaccuracies. The significance level of *p* < 0.05 was adopted. All calculations were performed using the statistical software Statistica version 13.

## 3 Results

### 3.1 Descriptive statistics

The study included 50 patients who underwent an elective TKA. The majority of patients were females (78%), mean age = 71.26 ± 7.88 years. A prevailing percentage of the patients had an elevated BMI according to the WHO standards: patients with normal weight – 6%, with overweight – 34%, with I degree obesity – 46% and with II degree obesity – 14%. A positive family history, with at least one parent affected with OA, was found in 34% of the patients. Knee pain appeared in the majority of almost half the patients (48%) not earlier than 5 years before. The majority of patients (56%) reported taking NSAIDs periodically due to the pain. NSAIDs were used constantly for the same reason by 24% of the patients. Severity of knee degeneration was assessed with a plain knee radiograph taken just before TKA. The Kellgren–Lawrence grade 5 scale was utilized for the pain assessment. The majority of patients (76%) revealed grade 4 OA, the highest degree of the degeneration involvement on the scale. Grades 0 and 1 were not observed among the patients.

### 3.2 Mutual relationships between concentrations of detected proteins

Mean values of the proteins obtained in three samples from the knee articulation were analyzed in each patient. The values were corrected according to the total protein concentration in the samples. MMP-13 was characterized by the biggest number of statistically significant correlations. All correlations were positive. The biggest strength of the linear relationship of the variables was between MMP-13 and TNF-α (Pearson’s *r*: 0.47; *p* < 0.001). A similar level of the relationship was found between MMP-13 and IL-6 (Pearson’s *r*: 0.46; *p* < 0.001), MMP-9 (Pearson’s *r*: 0.45; *p* < 0.001) and MMP-2 (Pearson’s *r*: 0.41; *p* < 0.001). The lowest correlation was between MMP-13 and MMP-1 (Pearson’s *r*: 0.34; *p* < 0.016). Moreover, a positive linear relationship was found between TNF-α and MMP-2 (Pearson’s *r*: 0.57; *p* < 0.001). No significant correlations were found for TGF-β1. In view of the fact that many results were beyond the range of reference, a further statistical analysis of IL-1β and MMP-3 was abandoned. An attempt to analyze a multivariate influence of the respective variables elucidated by means of metric data on protein concentrations was made. The results were not statistically significant. A set of Pearson’s correlation coefficients is shown in Table1.

**Table 1:** Pearson’s correlation between the investigated proteins

	MMP-1	MMP-2	MMP-9	MMP-13	IL-6	TNF-α	TGF-1β
MMP-1		0.17	0.17	**0.34**	0.23	0.26	0.05
MMP-2	0.17		0.11	**0.41**	0.19	**0.57**	0.18
MMP-9	0.17	0.11		**0.45**	0.07	0.26	0.03
MMP-13	**0.34**	**0.41**	**0.45**		**0.46**	**0.47**	0.11
IL-6	0.23	0.19	0.07	**0.46**		−0.10	−0.11
TNF-α	0.26	**0.57**	0.26	**0.47**	−0.10		0.08
TGF-1β	0.05	0.18	0.03	0.11	−0.11	0.08	

Values marked in bold indicate a statistically significant difference (*p* < 0.05).

### 3.3 Differences in protein distribution in different knee structures

**Figure 1:**
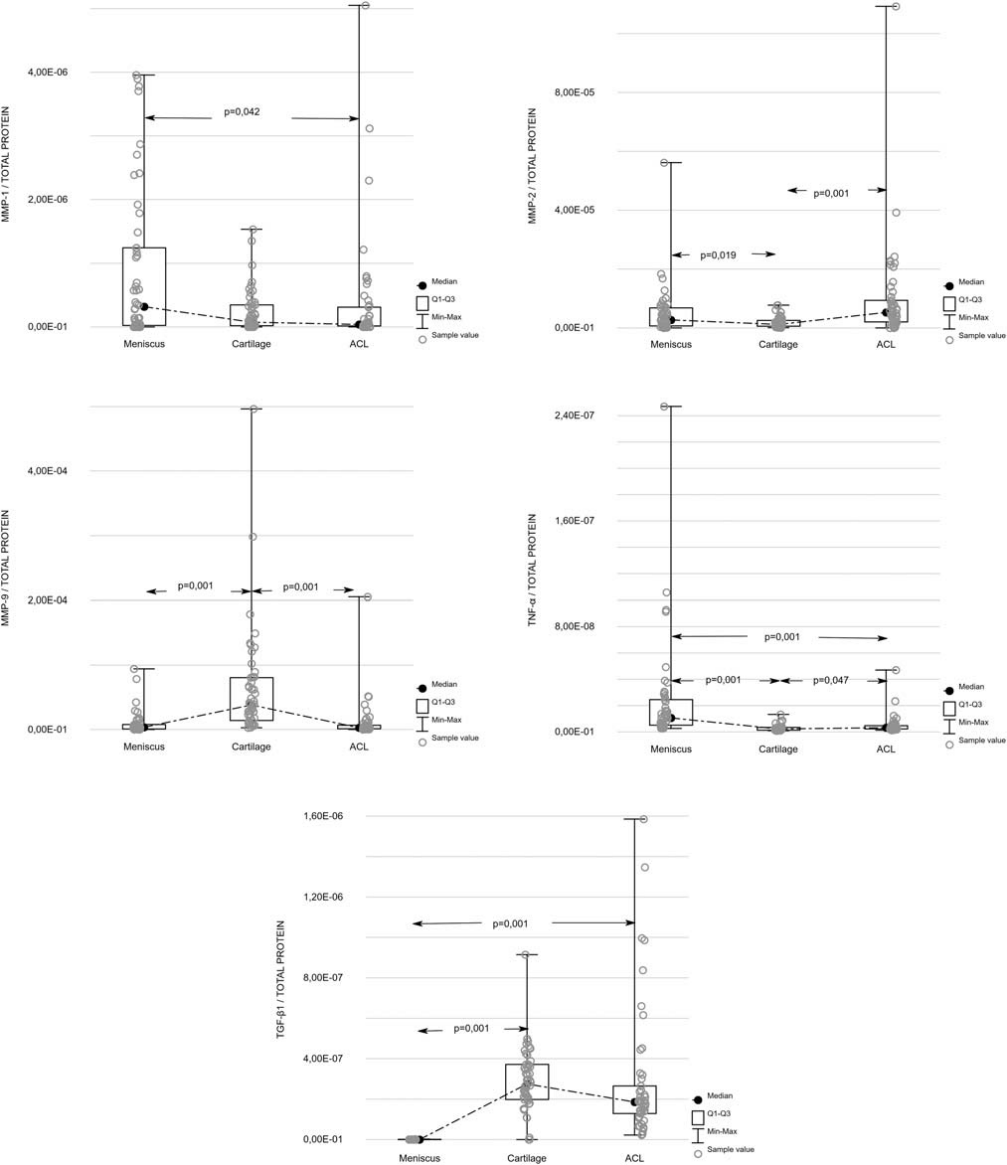
Concentration of investigated proteins (pg/ml) corrected with respect to overall protein concentration in anatomical structures (pg/ml).

Mean protein concentrations in different structures of the knee in relation to total protein in these structures are shown in Table 2. Statistically significant difference between respective structures was found in the case of MMP-1 (*p* < 0.05), MMP-2 (*p* = 0.001), MMP-9 (*p* = 0.001) and TNF-α (*p* = 0.001). These parameters were assessed once more with Tukey’s *post hoc* test, and the results are presented in Figure 1.

**Table 2:** Concentration of investigated proteins corrected with respect to overall protein concentration in anatomical structures

	*N*	Mean	Median	Lower quartile	Upper quartile	SD	*p*
MMP1 (pg/ml)/total protein (pg/ml)							
Meniscus	48	8.82 × 10^−7^	3.16 × 10^−7^	2.41 × 10^−8^	1.24 × 10^−6^	1.20 × 10^−6^	**0.035**
Cartilage	50	2.33 × 10^−7^	7.30 × 10^−8^	1.74 × 10^−8^	3.44 × 10^−7^	3.38 × 10^−7^	
ACL	49	3.64 × 10^−7^	3.92 × 10^−8^	1.62 × 10^−8^	3.08 × 10^−7^	8.96 × 10^−7^	
Mean	147	4.79 × 10^−7^	2.63 × 10^−7^	7.32 × 10^−8^	6.20 × 10^−7^	5.79 × 10^−7^	NA
MMP2 (pg/ml)/total protein (pg/ml)							
Meniscus	49	5.18 × 10^−6^	2.68 × 10^−6^	6.67 × 10^−7^	6.77 × 10^−6^	8.51 × 10^−6^	**0**.**001**
Cartilage	50	1.93 × 10^−6^	1.20 × 10^−6^	5.91 × 10^−7^	2.52 × 10^−6^	1.91 × 10^−6^	
ACL	49	9.81 × 10^−6^	5.24 × 10^−6^	1.97 × 10^−6^	9.33 × 10^−6^	1.67 × 10^−5^	
Mean	148	5.54 × 10^−6^	4.03 × 10^−6^	2.86 × 10^−6^	6.76 × 10^−6^	6.00 × 10^−6^	NA
MMP9 (pg/ml)/total protein (pg/ml)							
Meniscus	48	9.60 × 10^−6^	3.54 × 10^−6^	1.12 × 10^−6^	8.08 × 10^−6^	1.83 × 10^−5^	**0.001**
Cartilage	50	6.31 × 10^−5^	3.84 × 10^−5^	1.43 × 10^−5^	8.04 × 10^−5^	8.35 × 10^−5^	
ACL	49	1.13 × 10^−5^	2.88 × 10^−6^	1.30 × 10^−6^	6.96 × 10^−6^	3.07 × 10^−5^	
Mean	147	2.78 × 10^−5^	2.05 × 10^−5^	9.23 × 10^−6^	3.57 × 10^−5^	2.96 × 10^−5^	
MMP13 (pg/ml)/total protein (pg/ml)							
Meniscus	49	8.66 × 10^−6^	4.66 × 10^−6^	2.56 × 10^−6^	8.83 × 10^−6^	1.46 × 10^−5^	0.351
Cartilage	50	5.83 × 10^−6^	4.23 × 10^−6^	0.00 × 10^−1^	7.46 × 10^−6^	7.02 × 10^−6^	
ACL	49	5.50 × 10^−6^	3.43 × 10^−6^	2.19 × 10^−6^	4.98 × 10^−6^	9.72 × 10^−6^	
Mean	148	6.57 × 10^−6^	4.70 × 10^−6^	2.96 × 10^−6^	6.74 × 10^−6^	6.44 × 10^−6^	NA
IL6 (pg/ml)/total protein (pg/ml)							
Meniscus	49	3.92 × 10^−10^	0.00 × 10	0.00 × 10^−1^	0.00 × 10^−1^	1.72 × 10^−9^	0.814
Cartilage	50	9.13 × 10^−10^	0.00 × 10	0.00 × 10^−1^	0.00 × 10^−1^	4.15 × 10^−9^	
ACL	48	5.02 × 10^−10^	0.00 × 10	0.00 × 10^−1^	0.00 × 10^−1^	2.72 × 10^−9^	
Mean	147	6.05 × 10^−10^	0.00 × 10^−1^	0.00 × 10^−1^	3.25 × 10^−11^	2.82 × 10^−9^	NA
TNF-α (pg/ml)/total protein (pg/ml)							
Meniscus	50	2.29 × 10^−8^	1.06 × 10^−8^	5.06 × 10^−9^	2.46 × 10^−8^	3.95 × 10^−8^	**0.001**
Cartilage	50	2.97 × 10^−9^	2.30 × 10^−9^	1.39 × 10^−9^	3.45 × 10^−9^	2.44 × 10^−9^	
ACL	49	5.07 × 10^−9^	3.24 × 10^−9^	2.26 × 10^−9^	4.79 × 10^−9^	7.11 × 10^−9^	
Mean	149	1.03 × 10^−8^	5.28 × 10^−9^	3.45 × 10^−9^	1.12 × 10^−8^	1.42 × 10^−8^	NA
TGF-1β (pg/ml)/total protein (pg/ml)							
Meniscus	49	4.83 × 10^−10^	3.81 × 10^−10^	2.22 × 10^−10^	5.95 × 10^−10^	3.70 × 10^−10^	**0.001**
Cartilage	50	2.86 × 10^−7^	2.76 × 10^−7^	1.98 × 10^−7^	3.71 × 10^−7^	1.59 × 10^−7^	
ACL	49	2.91 × 10^−7^	1.85 × 10^−7^	1.28 × 10^−7^	2.66 × 10^−7^	3.32 × 10^−7^	
Mean	148	1.91 × 10^−7^	1.55 × 10^−7^	1.28 × 10^−7^	2.21 × 10^−7^	1.23 × 10^−7^	NA

Values marked in bold indicate a statistically significant difference (*p* < 0.05).

### 3.4 Impact of demographic and ontogenetic parameters

The parameters such as sex, age, BMI, the use of NSAID, tobacco smoking and OA in the family history have potential impact on mean protein concentration, as well as protein concentration in different structures of the knee (in relation to total protein).

**Figure 2:**
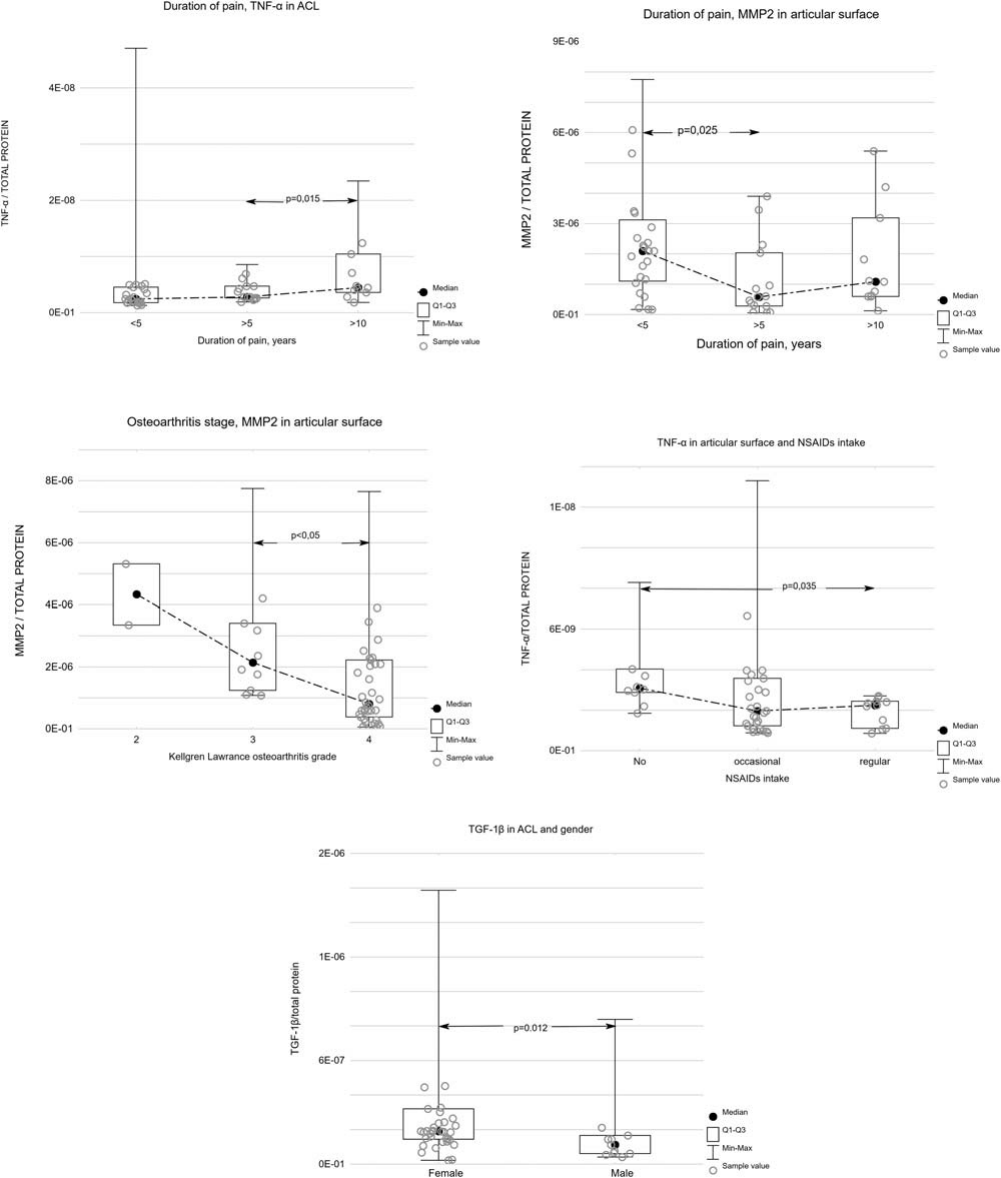
Demographic medical history data and evaluation of OA stage. Concentration of investigated proteins (pg/ml) corrected with respect to overall protein concentration in anatomical structures (pg/ml).

In terms of sex, the only statistically significant difference (*p* = 0.012) in protein concentration was in the case of TGF-β1 in the samples obtained from the ACL. The concentration of TNF-α in the ACL was higher in females: 5.51 × 10^−0.9^ ± 1.25 × 10^−0.7^, while in males it was 3.56 × 10^−0.9^ ± 5.10 × 10^−0.8^. A negative linear relationship was observed between the age and the mean MMP-1 (Pearson’s *r*: −0.91; *p* = 0.001) and also TNF-α (Pearson’s *r*: −0.88; *p* < 0.020). The TNF-α concentration was statistically significantly lower in the cartilage of the articular surface among the patients who took NSAIDs periodically (1.96 × 10^−0.9^ ± 1.89 × 10^−10^, compared to the TNF-α concentration among the patients who did not take NSAIDs, 4.00 × 10^−7.12^ ± 1.2 × 10^−10^; *p* = 0.035). There were no statistically significant differences in detected protein concentrations, in the patients with a positive family history of degenerative disease of the locomotor system, in the patients with different BMI categories according to the WHO and in smokers. The results of the analysis are shown in Figure 2.

### 3.5 Protein concentration and functional parameters

Clinical scales were compared against mean protein concentration values in the knee deriving from three samples in each patient. The values were corrected according to the total protein concentration. A higher result on the WOMAC scale (signifying a better clinical condition) coexists with a lower level of MMP-1 (Pearson’s *r*: −0.82; *p* = 0.041) and TNF-α (Pearson’s *r*: −0.84; *p* = 0.038). There were no statistically significant correlations regarding the remaining scales and the VAS. The results of the analysis are presented in Table 3.

**Table 3:** Pearson’s correlation between the investigated proteins and the clinical scores

	HHS	KSS-clinical	KSS-function	VAS	KOOS	WOMAC	SF-36
MMP-1	0.72	0.78	0.61	−0.07	0.77	**−0**.**82**	0.43
MMP-2	0.17	−0.27	0.04	0.40	−0.57	0.59	−0.38
MMP-9	0.43	−0.05	0.50	0.11	−0.09	0.14	−0.42
MMP-13	0.43	0.10	0.67	−0.15	0.29	−0.24	−0.24
IL-6	0.43	0.01	0.47	0.09	0.02	0.02	−0.44
TNF-α	0.56	0.64	0.60	−0.13	0.78	**−0**.**84**	0.47
TGF-1β	0.33	−0.04	0.11	0.26	−0.19	0.21	−0.57

Values marked in bold indicate a statistically significant difference (*p* < 0.05).

### 3.6 Protein concentration – severity of the disease and pain

The decrease in MMP-2 in the cartilage of the articular surface corresponded with the severity of radiological OA on the Kellgren–Lawrence scale. The significance of the observed differences was confirmed by the Kruskal–Wallis test and then by Tukey’s *post hoc* test, in which a significant difference between a group of patients with III degree (2.80 × 10^−0.6^ ± 2.04 × 10^−0.6^) and a group with IV degree of severity of OA (1.57 × 10^−0.6^ ± 1.76 × 10^−0.6^) (*p* < 0.05) was observed. Among patients who had suffered from pain for less than 5 years, a higher concentration of MMP-2 was noted in the cartilage of the articular surface than among those who had suffered from pain for more than 5 years. Moreover, in the group of patients who had suffered from pain for less than 5 years (4.77 × 10^−0.9^ ± 9.31 × 10^−0.9^), a lower concentration of TNF-α in the ACL was detected than in patients suffering from pain lasting for over 10 years (7.21 × 10^−0.9^ ± 6.27 × 10^−0.9^). The results are presented in Figure 2.

## 4 Discussion

Both the development and progression of OA are affected by numerous molecular factors that are engaged in the inflammatory response. At this level, our knowledge concerning processes and mechanisms occurring in the course of OA relies mainly on the analyses of the synovial fluid and epiphyseal cartilage. The pathogenesis of the process and thus treatment protocols are set on that basis. Molecular alterations occurring within each of knee joint structures might aggravate the course and severity of pathological lesions observed in OA.

Limited scientific data are available regarding the content of particular molecules within the surgical specimen, especially in terms of the control group. The aforementioned studies are usually based on the analyses of synovial fluid. The analysis allowed identification of the differences between the activities of specific processes and mediators in various structures of the osteoarthritic knee joint. It should be stressed that, to date, the analyses concerned the assessment of the synovial fluid and cartilage. In our study, the investigation was expanded to include ACL and meniscus. Unfortunately, the literature contains no such research reports.

The highest concentrations in the ACL were observed for TNF as well as MMP-2 and MMP-3; in the meniscus, they were noted for TNF, MMP-1 and MMP-13. The presence of the aforementioned molecules suggests type I collagen degeneration, along with the inflammatory process localized also in this particular joint component in the course of OA. The most important factors within the articular cartilage were, in turn, IL-6, TGF-β and MMP-9. IL-6 and TGF-β influence the activation of Th17 lymphocytes, which are indicative of chronic inflammatory processes. The presence of the aforesaid transmitters suggests that inflammation localized within all of the investigated knee components.

The aforesaid values have no reference to the normal condition (they cannot be assessed) and represent mere arithmetic differences in the severity of the pathological condition. Because of the complexity of the observed mechanisms, no single treatment method targeted at one selected process exists. We should aim at searching for therapies that regulate numerous pathways engaged in both the degeneration of the joint structures and the severity of the inflammatory process.

The proinflammatory profile of the synovial fluid may be correlated with pain severity in OA. Leung et al. [15] investigated the synovial fluid profiles in 70 patients. The aspirates were tested for IL-1β, IL-6, IL-8, TNF-α, C-terminal telopeptides of type I collagen (CTXI), C-telopeptide of type II collagen (CTXII) and urinary CTXII levels. According to the results, IL-6, IL-8 and IL-1β concentrations substantially influenced pain severity during movement, but not at rest. No significant correlations in the WOMAC scale were observed for the aforementioned cytokine concentrations. This is in contrast to the results of our work, in which we proved a negative correlation between better clinical outcome measured with WOMAC and TNF-α and MMP-1 concentrations.

However, TNF-α levels were positively correlated with pain severity at rest and during movement, as assessed with both Likert and WOMAC scales. In our group, there were no correlations between investigated cytokines, metalloproteinases and the VAS scale. Orita et al. [20] examined the synovial fluid of 47 patients to investigate the relationship between the presence of the following proinflammatory cytokines: TNF-α, IL-6 and nerve growth factor, and OA severity graded with radiologic Kellgren–Lawrence and clinical WOMAC scales. The results showed that TNF-α concentration did not affect the grade of OA lesions assessed with the Kellgren–Lawrence scale but was correlated with disease severity measured with both the total WOMAC scale and its three subscales. The level of IL-6 showed correlation with lesion severity assessed by radiologic means. In the WOMAC scale, the correlation with IL-6 was only visible in the stiffness subscale. One of the studies [21] aimed to compare biochemical profiles of the synovial fluid in patients with meniscus injuries. The authors discovered a positive association between pain severity and IL-6, monocyte chemotactic protein-1, macrophage inflammatory protein-1β and interferon gamma concentrations. They made an observation that increased levels of the aforesaid cytokines occur in symptomatic meniscus injuries. The levels were considerably lower in the case of asymptomatic injuries, comparable to healthy, asymptomatic knee joint. It is therefore possible that increased levels of the aforementioned cytokines are responsible for the emergence of pain symptoms. In the study by Denoble et al. [22], the IL-1β concentration was linked with the severity of radiological symptoms assessed with the Kellgren–Lawrence scale. In our study, the decrease in MMP-2 in the articular surface corresponded with the severity of OA and among patients who had suffered from pain for less than 5 years a higher concentration of MMP-2 was noted in the articular surface than among those who had suffered from pain for more than 5 years. These results may suggest that some inflammatory parameters decrease along with the progression of the disease.

A relationship between increased IL-1β expression and increased MMP-13 content has been proven as well. According to the research by Billinghurst et al. [23], the increased activity of chondrocyte collagenases is responsible for type II collagen disintegration within articular cartilage of the femoral condyles collected from patients with degenerative arthritis. The authors investigated the activity of the following proteases: MMP-1, MMP-8 and MMP-13. The results indicated the particularly important role of MMP-13 in the process.

The role of TGF-β in the etiology of degenerative arthritis has not been unambiguously defined yet [24]. According to the study by Jeffries et al., genes coding TGF-β- and TNF-dependent signaling pathways exhibit methylation differences in both the subcartilaginous bone and the cartilage of patients suffering from OA [25]. The research on animal models with the use of anti-TGF-β antibodies has proved their role in reducing the severity of degenerative lesions in mice [26]. However, the results of research with large amounts of antibodies against TGF-β suggest that certain level of this molecule is essential to maintain the integrity of both cartilage and bone tissues. Other studies also confirmed the significance of TGF-β-dependent pathway as a potential target for OA treatment [24].

The availability of studies that assess concentrations of the proinflammatory factors within meniscus and ACL of patients with OA is negligible.

The aforesaid results, based on the assessments of the synovial fluid and tissues collected from the knee joint, depict the complexity of molecular processes underlying both the etiology and progression of OA. Likewise, in our study, increased concentrations of proinflammatory cytokines and chemokines, as well as their different levels within diverse components of the knee joint (articular surfaces, menisci and ACL), were shown. Current treatment methods for OA generally do not stop disease progression, but primarily act symptomatically. Numerous patients will finally experience such disease progression that they will require arthroplasty of the diseased joint. Identifying signaling pathways and molecular particles engaged in OA and their correlations with the patient’s clinical condition brings new therapeutic possibilities to stop/slow down the disease progression on the molecular level.

Current treatment methods for OA generally do not stop disease progression. Articular arthroplasty remains the ultimate treatment option for numerous patients. Identifying signaling pathways and molecular particles engaged in OA and their correlations with the patient’s clinical condition/medical history brings new therapeutic possibilities. The study demonstrated that changes in the cytokine, chemokine and metalloproteinase levels in the advanced OA occur not only within the articular cartilage but also other structures of the knee joint – the meniscus and the ACL.

In addition, each of the joint structures was dominated by different activity of diverse molecular pathways. This indicates the multi-complexity of the degenerative joint disease on the molecular level. Therefore, no single treatment method targeted at one selected process exists. We should aim at searching for methods/therapies that regulate numerous pathways engaged in the degeneration of joint structures.
